# Upregulated TSG-6 Expression in ADSCs Inhibits the BV2 Microglia-Mediated Inflammatory Response

**DOI:** 10.1155/2018/7239181

**Published:** 2018-11-21

**Authors:** Yang Hu, Gaigai Li, Ye Zhang, Na Liu, Ping Zhang, Chao Pan, Hao Nie, Qi Li, Zhouping Tang

**Affiliations:** ^1^Department of Neurology, Tongji Hospital, Tongji Medical College, Huazhong University of Science and Technology, Wuhan Hubei 430030, China; ^2^Department of Neurology, The First Affiliated Hospital of Chongqing Medical University, Chongqing 400016, China

## Abstract

**Objectives:**

The microglial cells are immune surveillance cells in the central nervous system and can be activated during neurological disorders. Adipose-derived stem cells (ADSCs) were reported to inhibit the inflammatory response in microglia by secreting proteins like tumor necrosis factor-inducible gene 6 protein (TSG-6). We aim to explore the mechanisms and the associated microRNAs.

**Methods:**

ADSCs were cultured and TSG-6 expression was evaluated. ADSCs were cocultured with lipopolysaccharide- (LPS-) induced BV2 microglia and the supernatant was harvested for detecting cytokines. The total RNA was extracted and sequenced by high-throughput sequencing. MicroRNA profiles were compared between two treatment groups of ADSCs. A comprehensive bioinformatics analysis and confirmation experiments were performed to identify the microRNAs targeting at TSG-6.

**Results:**

We found that ADSCs could secrete TSG-6 to inhibit the proinflammatory cytokines, including interleukin-1 beta and interleukin-6, and tumor necrosis factor alpha (TNF*α*), produced by LPS-induced microglia-mediated inflammatory response. Bioinformatics analysis showed a total of 35 microRNAs differentially expressed between the two groups of ADSCs, and miR-214-5p was identified as a regulator of TSG-6 mRNA.

**Conclusion:**

Following a treatment with TNF*α*, ADSCs can regulate the inflammatory response in LPS-activated BV2 microglia by upregulating TSG-6 expression, which itself is under the negative control of miR-214-5p.

## 1. Introduction

Microglia are residential macrophages in the central nervous system during embryonic development. They mainly mediate the innate immune response and maintain homeostasis under physiological conditions [1, 2]. Once exposed to the inflammatory stimuli or mediators, microglia will be activated immediately and will release various cytokines, including interleukin-1 beta (IL-1*β*), tumor necrosis factor alpha (TNF*α*), and inducible nitric oxide synthase (iNOS) [3]. Recent evidences have indicated that microglial dysfunction is associated with stroke and various neurodegenerative diseases like Alzheimer's disease (AD), Parkinson's disease (PD), and amyotrophic lateral sclerosis (ALS) [4]. At the same time, activated microglia have been considered as a therapeutic target for these diseases. Mesenchymal stem cells (MSCs) can alleviate inflammation and have been suggested as a promising treatment in neurological disorders. Several studies have shown that mesenchymal stem cells can inhibit the inflammatory response in lipopolysaccharide- (LPS-) activated BV2 microglia [5].

Adipose-derived stem cells (ADSCs) are mesenchymal stem cells derived from adipose tissue and are similar to bone marrow derived mesenchymal stem cells [6, 7]. Numerous studies have shown that transplanted ADSCs can promote angiogenesis and tissue regeneration. The involved mechanisms mainly include differentiation into host damaged tissue and secretion of a variety of soluble factors such as angiotensin, cytokines, and neurotrophic factors [8]. The tumor necrosis factor alpha (TNF*α*) can induce the expression of an important anti-inflammatory protein and tumor necrosis factor-inducible 6 (TSG-6). Song et al. found that TSG-6 released from intraperitoneally injected adipose tissue-derived mesenchymal stem cells could ameliorate inflammatory bowel disease by inducing M2 macrophage switch in mice [9].

MicroRNAs are noncoding single-stranded RNA molecules of about 19-25 nucleotides in length and are highly conserved among species [10]. Mature microRNAs in vivo can inhibit mRNA encoding and protein translation by directly cleaving mRNA, or decomposing mRNA through deadenylation. MicroRNAs have recently been reported to regulate the immune response [11, 12].

Our study aimed to assess the immune-modulatory effects of ADSCs-produced TSG-6 on LPS-induced microglia and explore the associated microRNAs.

## 2. Materials and Methods

### 2.1. Ethical Statement

Clean grade, female Sprague-Dawley rats, aged three to four weeks and weighing 100-120 g, were provided by the Experimental Animal Center of Tongji Medical College (Wuhan, Hubei Province, China). All experimental protocols were in accordance with the Regulations for the Administration of Affairs Concerning Experimental Animals, directed by the Chinese Ministry of Science and Technology. Both the experimenter and person assigning subjects to groups were blinded throughout the experimental course.

### 2.2. Isolation and Culturing of Rat ADSCs and BV2 Microglia

ADSCs were obtained from adipose tissue of Sprague-Dawley rats (100–120 g) provided by the Animal Laboratory Center of Tongji Medical College of Huazhong University of Science and Technology as previously described [13]. Briefly, the adipose tissue was dissected from the inguinal fat depots of rats and wash with sterile phosphate-buffered saline (PBS; pH 7.4; Hyclone; Thermo Scientific, Logan, UT, USA) to remove red blood cells and debris. The cleaned tissue was then digested by 0.1% collagenase I (Invitrogen, Carlsbad, CA, USA) for 40 min at 37°C. Dissociated cells were harvested and plated on a 25 cm^2^ tissue culture dish (Corning, Lowell, MA, USA) and incubated at 37°C and 5% CO_2_. Nonadherent cells were removed by replacing the culture medium after 24 h, and the medium was replaced every two to three days. At 80-90% confluence, cells were passaged by detaching adherent cells with a trypsin-EDTA solution (0.25% trypsin and 0.02% EDTA; Genom) and resuspended and plated in Dulbecco modified Eagle medium F12 (DMEM/F12; Hyclone) supplemented with 1% penicillin/streptomycin (Solarbio), 1% l-glutamine (Genom), and 10% fetal bovine serum (FBS) (Gibco). The third to fifth passage cells were used in this study.

The BV2 cell line was purchased from the American Type Culture Collection. It was maintained in DMEM/F12 (Hyclone) with 10% FBS (Gibco) and 1% penicillin-streptomycin (Solarbio) at 37°C in a humidified atmosphere containing 5% CO2.

### 2.3. Flow Cytometry Analysis of Rat ADSCs

The third-passage rat ADSCs were tested by flow cytometry analysis. Briefly, the adherent cells were harvested by trypsinization, centrifuged at 800 rpm for 6 min, washed with sterile phosphate-buffered saline, and resuspended. Cells were stained with specific fluorescein isothiocyanate-conjugated (FITC) monoclonal antibodies against rat CD29, CD45, and CD34 for 45 min at 4°C (Supplement Table S1). For the negative control, an irrelevant antibody of the same isotype was used. Finally, the cells were analyzed using a FACS Calibur cytometer (BD).

### 2.4. Treatment with TNF*α*

ADSCs were treated with recombinant rat TNF*α* (Peprotech USA) at the concentration of 10 ng mL^−1^ for 16-18 h. In brief, ADSCs were plated in six-well plates (Corning) at the density of 2 × 10^5^ cells per well. After incubation with DMEM F12 containing 10% heat-inactivated FBS for 24 h, the medium was changed to serum-free DMEM F12 containing 10 ng mL^−1^ TNF-*α* (Peprotech). After incubation for 18 h, the cells were harvested and used for further analyses.

### 2.5. Interference of Adipose Stem Cells with TSG-6-siRNA and Anti-miR Treatments

ADSCs were transfected with TSG-6-siRNA and control-siRNA respectively. Briefly, at 80-90% confluence, ADSCs were seeded in six-well plates at the density of 4 × 10^5^ cells per well in 2 mL antibiotic-free complete medium. After adhering for about 24 h in a suitable environment, the ADSCs were transfected with TSG-6-siRNA or control-siRNA (all siRNA sequences were purchased from RiBo company, Guangzhou) using lipofectamine 2000 reagent (Invitrogen, USA) and Opti-MEM I Reduced Serum Medium (Gibco). The ADSCs were incubated at room temperature for about 6 h and treated with TNF*α* subsequently.

The same protocol was applied to anti-miR treatments and the ADSCs were transfected with the microRNA mimic, inhibitor, or respective control at the final concentration of 100uM (all mimic, inhibitor and control sequences were purchased from RiBo company, Guangzhou) using lipofectamine 2000 reagent. The siRNA and miR sequences were shown in Supplementary File.

### 2.6. ADSCs/ BV2 Microglia Cocultures

ADSCs were cocultured with BV2 microglia in a six-well transwell system (0.4 *μ*m pore size membrane; Corning). A total of 5 × 10^5^ BV2 microglia were seeded in the lower chamber and the upper chamber was seeded with one of the following cells or culture medium: (1) control medium; (2) 1.0 × 10^5^ activated ADSCs transfected with TSG-6-siRNA; (3) 1.0 × 10^5^ activated ADSCs transfected with control-siRNA; (4) LPS at the concentration of 100 ng mL^−1^. The BV2 microglia were then harvested and prepared for the cytokines test including TNF*α*, IL-1*β*, IL-6, and iNOS.

### 2.7. MicroRNA Sequencing and Expression Detection

The expression of genome-wide microRNAs was detected by high-throughput sequencing. Total RNA was extracted from ADSCs treated with TNF*α* and control medium. Initially, 1 *μ*g of 18-30 nucleotide RNA was screened to construct a cDNA sequencing library using TruSeq® Small RNA Sample Prep Kit (Illumina). After end polishing, A-tailing, adapter ligation, and size selection, the qualified cDNA library of moderate length, at a final concentration of 10 pM, was sequenced according to manufacturer's instruction using Illumina HiSeqTM 2500. Poor quality data was removed from the raw reads to get clean data, which was subsequently compared to all the mature rat miRNA sequences in miRBase database (Version21). Then the miRNA expression was calculated and normalized using the formula: the number of reads per million (RPM) equals number of reads mapping to (miRNA/number of reads in clean data) *∗* 10^6^.

### 2.8. Comparison Analysis

To compare the difference in known miRNAs expression between two groups of ADSCs, the log2 ratio was calculated and heat mapping of clustering analysis was drawn, where log2 (Fold Change) equals the absolute value of log2 (normalized expression value in sample 2/normalized expression value in sample 1). Differential miRNA screening criteria: in the experiment, the results showed the direction and extent of change, log2 (Fold Change) absolute value greater than or equal to 1, P <0.01 for the significant difference, log2 (Fold Change) absolute value greater than or equal to 1, P<0.05 for the general significant difference, P≥0.05 for the general difference. We refer to the published literature for p value calculation [14].

### 2.9. Quantitative Real-Time PCR

Total RNA was extracted from ADSCs and BV2 microglia using trizol (Introvergen) according to the manufacturer's protocol. The RNA was used for reverse transcription using RevertAid™ First Strand cDNA Synthesis Kit (Fermentas). The RT-PCR was performed using first SYBR Green/Fluorescein qPCR Master Mix (Thermo Bio) with primers for GADPH, TNF*α*, IL 1*β*, IL6, TNFa, and iNOS. The amplification conditions were as follows: 95°C for 3 min, followed by 40 cycles of 95°C for 5s, 60°C for 30 s and 72°C for 40s, 95°C for 10s using Bio-rad iQTM5 (Bio-rad). Data were analyzed using the 2-ΔΔCt method, the same protocol for mir214-5p detection except for the primers and amplification conditions. All the gene primers and amplification conditions were shown in Supplementary File.

### 2.10. ELISA

To evaluate TSG-6 protein production, single-cell suspensions were adjusted to a concentration of 1x10^6^ mL^−1^ and seeded in six-well plates. Cell-free supernatants were collected and assayed using an ELISA kit (Biovalue, California, United States) according to manufacturer's instructions. Briefly, standard, control, and test samples were added to the wells and incubated for appropriate time with 50 *μ*L primary antibody and 10 *μ*L conjugate. After washing, 50 *μ*L substrate solution was added to each well for 15 min at room temperature in the dark. Finally, the 50 *μ*L stop solution was added and the OD value at the wavelength of 450 nm and 570 nm in each well was measured within 30 min using a microplate reader.

The cocultured BV2 microglia were seeded in the lower chamber of transwell system. To evaluate cytokines production, cell-free supernatants were collected and assayed using an ELISA kit (Elabscience, Hubei, China) of according to manufacturer's instructions. Briefly, standard, control, and test samples were added to the wells and incubated for appropriate time with 100 *μ*L primary antibody and 90 *μ*L conjugate. After washing, 50 *μ*L substrate solution was added to each well for 15 min at room temperature in the dark. Finally, the 50 *μ*L stop solution was added and the OD value at the wavelength of 450 nm in each well was measured within 30 min using a microplate reader.

### 2.11. Statistical Analysis

Student's t-test was used for comparison between two groups and One-Way ANOVA was used for comparison among three or more groups using SPSS 20.0 software. All data from at least three independent experiments using three different cell cultures were expressed as the mean ± standard deviation; p < 0.05 was considered statistically significant.

## 3. Results

### 3.1. Isolation and Characterization of Rat ADSCs

**Figure 1:**
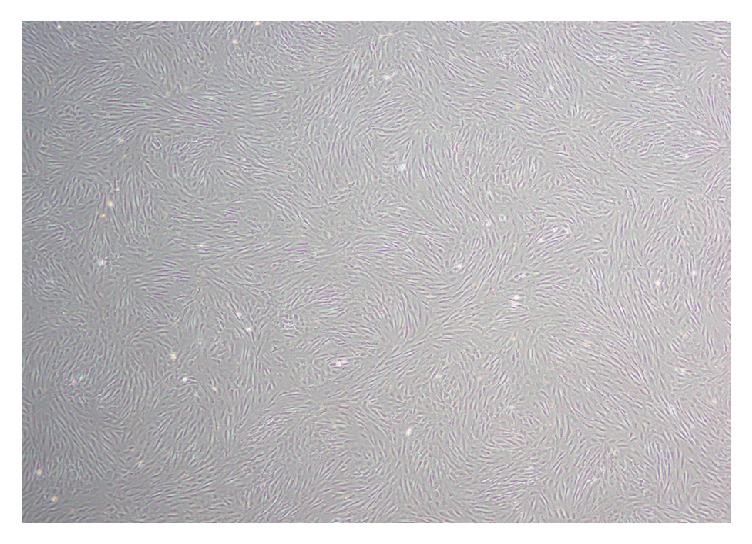
The third-passage ADSCs: phase contrast image of ADSCs (40x) revealed a typical spindle-like morphology.

**Figure 2:**
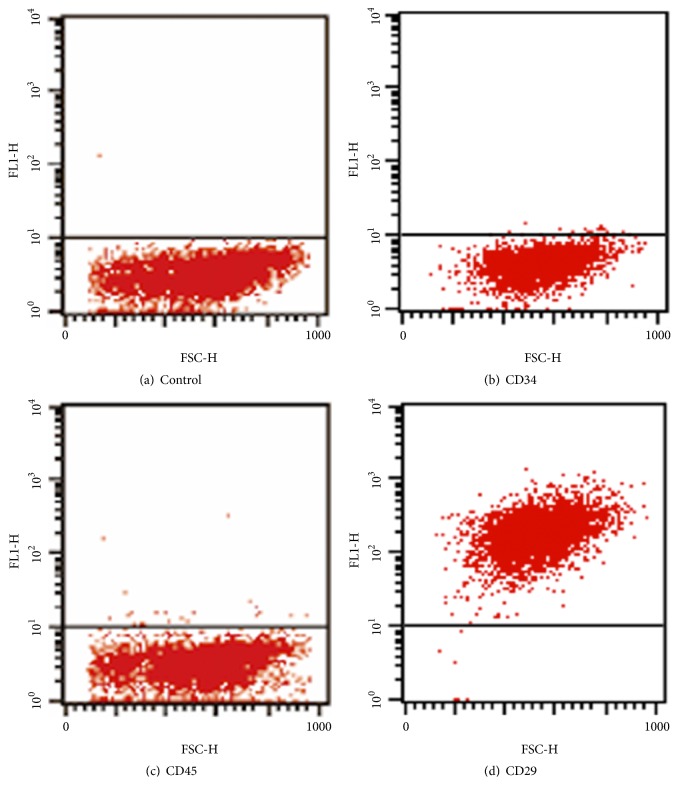
Flow cytometry of cultured ADSCs: the horizontal axis represents FSC-height, and the vertical axis represents FL1-H. Results are representative of three independent experiments using three different ADSCs cultures.

Rat ADSCs were isolated and planted on tissue culture plates. They adhered to the plastic surfaces and exhibited a spindle-shaped morphology at first, and then they transformed into homogeneously flat, fibroblast-like cells. The cell culture was subcultured every three to four days (Figure 1). To further characterize rat ADSCs, cell surface markers of the third-passage cells were examined by flow cytometry. Flow cytometry analysis revealed that the cells showed high expression of the characteristic MSC marker CD29 (99.80%) and low expression of the hematopoietic markers CD34 and CD45 (0.56% and 0.59%, resp.) (Figure 2).

### 3.2. The TSG-6 Expression Was Upregulated in ADSCs Treated with TNF*α*

**Figure 3:**
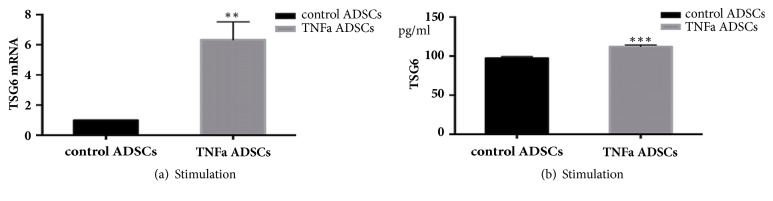
The TSG-6 expression was upregulated in ADSCs treated with TNF*α*. Results are representative of three independent experiments using three different ADSCs cultures. (a) The expression of TSG-6 was increased sixfold in the treatment group, *∗∗*P<0.01, significant differences from respective control group. (b) TSG-6 protein was slightly increased in the treatment group, *∗∗∗*P<0.001, significant differences from respective control group.

TSG-6 mRNA and protein levels were significantly higher in ADSCs treated with TNF*α* (Figure 3). Results showed that the expression of TSG-6 RNA was increased sixfold in the treatment group as compared to the control. These observations were supported by a slight increase in TSG-6 protein from the control treatment.

### 3.3. TSG-6-siRNA Effectively Interfere with the TSG-6 Expression in ADSCs Treated with TNF*α*

**Figure 4:**
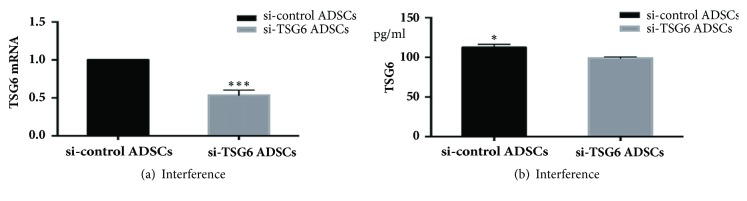
TSG-6-siRNA effectively interferes with the TSG-6 expression in ADSCs treated with TNF*α*. Results are representative of three independent experiments using three different ADSCs cultures. TSG-6 mRNA (a) and TSG-6 protein (b) expression were significantly decreased in ADSCs transfected with TSG-6-siRNA as compared to control-siRNA, *∗*P<0.05, *∗∗∗*P<0.001.

After transfection with either TSG-6-siRNA or control-siRNA, TSG-6 mRNA and protein levels in TNF*α*-treated ADSCs were monitored. qPCR data showed a significant decline in TSG-6 expression in ADSCs transfected with TSG-6-siRNA as compared to control-siRNA (Figure 4), which was consistent with the protein level, indicating that the interference was effective (Figure 4).

### 3.4. TNF*α*-Treated ADSCs Inhibit LPS-Induced Inflammatory Responses of BV2 Microglia through the Secretion of TSG-6

**Figure 5:**
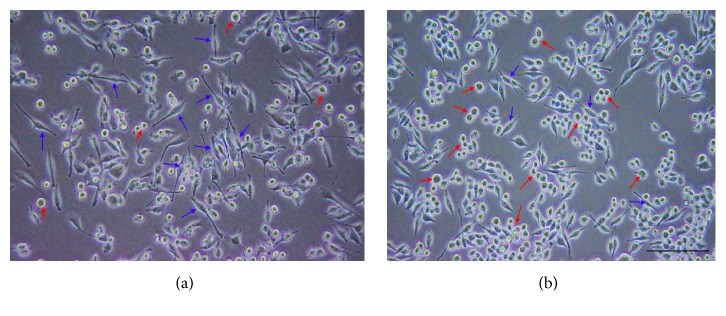
BV2 microglia morphologies before (a) and after LPS induction (b). Green arrows indicate ramified morphologies with elongated cellular projections; red arrows indicate activated morphologies with resorbed projections.

**Figure 6:**
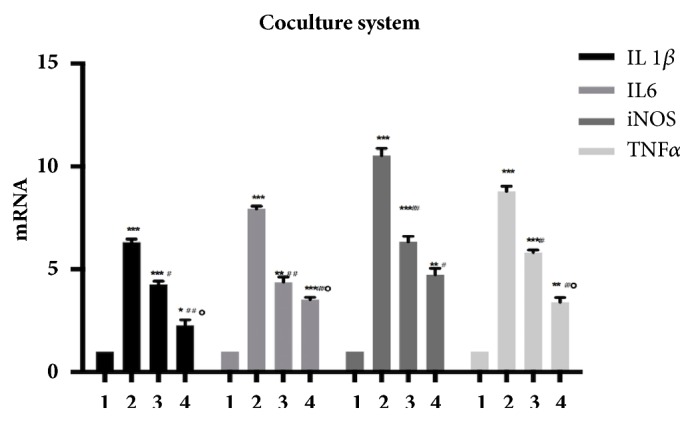
TNF*α*-treated ADSCs inhibit LPS-induced inflammatory responses of BV2 microglia through the secretion of TSG-6. Results are representative of three independent experiments using three different cell cultures. The mRNA levels of IL1*β*, IL6, iNOS, and TNFa were detected. There are four treatment groups in total, group 1 (BV2 microglia), group 2 (BV2 microglia+LPS), group 3 (BV2 microglia+LPS+TSG-6-siRNA ADSCs), and group 4 (BV2 microglia+LPS+control-siRNA ADSCs). Comparison with group 1 (BV2 microglia), *∗*P<0.05, *∗∗*P<0.01, and *∗∗∗*P<0.001; with group 2 (BV2 microglia+LPS), #P<0.05, ##P<0.01, and ###P<0.001; with group 3 (BV2 microglia+LPS+TSG-6-siRNA ADSCs), ∘P<0.05 and group 4 (BV2 microglia+LPS+control-siRNA ADSCs).

**Figure 7:**
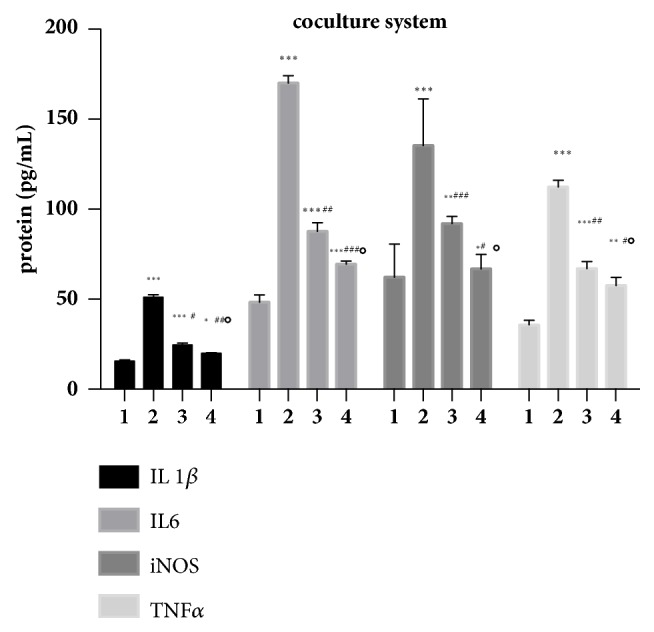
TNF*α*-treated ADSCs inhibit LPS-induced inflammatory responses of BV2 microglia through the secretion of TSG-6. Results are representative of three independent experiments using three different cell cultures. The protein levels of IL1*β*, IL6, iNOS, and TNFa were detected. There are four treatment groups in total, group 1 (BV2 microglia), group 2 (BV2 microglia+LPS), group 3 (BV2 microglia+LPS+TSG-6-siRNA ADSCs), and group 4 (BV2 microglia+LPS+control-siRNA ADSCs). Comparison with group 1 (BV2 microglia), *∗*P<0.05, *∗∗*P<0.01, and *∗∗∗*P<0.001; with group 2 (BV2 microglia+LPS), #P<0.05, ##P<0.01, and ###P<0.001; with group 3 (BV2 microglia+LPS+TSG-6-siRNA ADSCs), ∘P<0.05 and group 4 (BV2 microglia+LPS+control-siRNA ADSCs).

The microglia immediately transformed from the ramified form with elongated cellular projections (Figure 5(a)) to the fully active phagocytic form with resorbed projections (Figure 5(b)) once LPS was added to the culture medium. Further, results from qPCR and ELISA assay demonstrated that the expression of interleukin 1*β*, interleukin 6, iNOS, and TNF*α* was significantly elevated in LPS-induced BV2 microglia. All four cytokines in BV2 microglia were inhibited when cocultured with ADSCs for 6 h using a transwell coculture system. In addition, IL-1*β*, IL-6, iNOS, and TNF*α* were significantly decreased in TSG-6-siRNA transfected ADSCs as compared to the control-siRNA transfected group (Figures 6 and 7).

### 3.5. miRNA Expression Profile of ADSCs with and without TNF*α* Treatment

**Figure 8:**
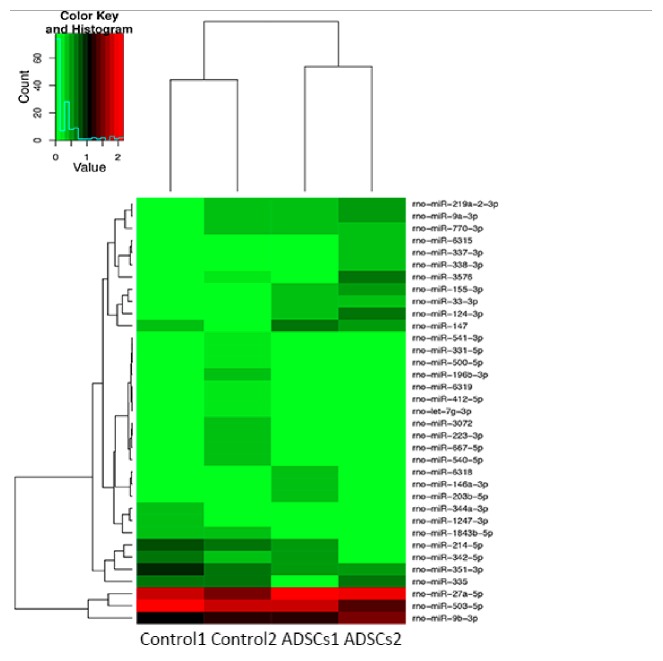
The heat-map diagram of miR cluster analysis in ADSCs with and without TNF*α* treatment. Red indicates that the miRNA is highly expressed in the sample, and green indicates that the expression of miRNA in the sample is low. Each row represents microRNA, and each column represents a sample.

The cDNA sequencing library was satisfactory for the next sequencing procedure. The raw reads and cleaned data were also both well qualified. Through difference analysis and cluster analysis, 35 microRNAs were found to be differentially expressed between TNF*α*-treated and untreated ADSCs, of which 19 microRNAs were downregulated in TNF*α*-treated ADSCs (Table 1; Figure 8; Supplement Table S2).

### 3.6. miR-214-5p Was Upregulated in ADSCs Treated with TNF*α*

**Figure 9:**
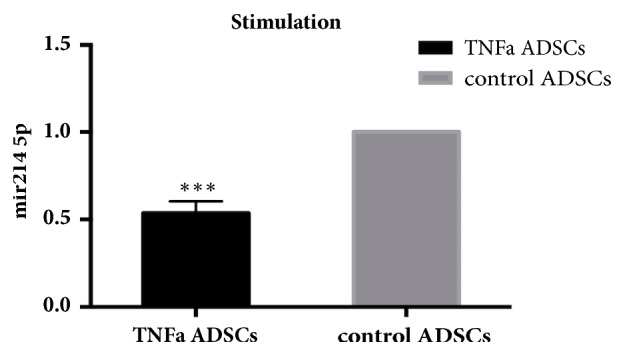
miR-214-5p was downregulated regulated in ADSCs treated with TNFa. Results are representative of three independent experiments using three different ADSCs cultures. miR-214-5p levels were 50% lower when ADSCs were treated with TNF*α* compared to the control group, *∗∗∗*P<0.001.

qPCR was performed in order to validate the differential expression of miR-214-5p between TNF*α*-treated and untreated ADSCs. The outcome was consistent with the data obtained from the high-throughput sequencing, showing that miR-214-5p levels were 50% lower when ADSCs were treated with TNF*α* compared to the control group (Figure 9).

### 3.7. miR-214-5p Negatively Regulates the Immunosuppressive Capacity of ADSCs

**Figure 10:**
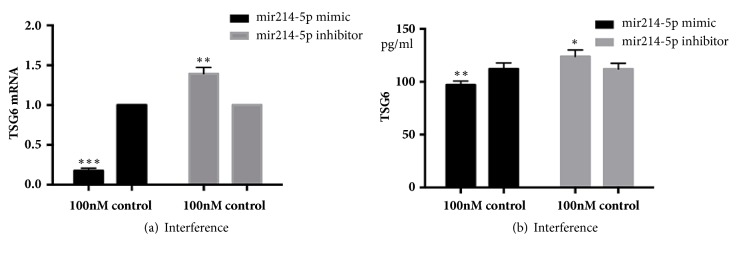
miR-214-5p negatively regulates the immunosuppressive capacity of ADSCs. Results are representative of three independent experiments using three different ADSCs cultures. TSG-6 mRNA (a) and TSG-6 protein (b) were significantly downregulated in ADSCs transfected with a miR-214-5p mimic compared to the mimic control and increased compared to the control when ADSCs were transfected with a miR-214-5p inhibitor, *∗*P<0.05, *∗∗*P<0.01, and *∗∗∗*P<0.001.

To validate our results, an experiment was carried out whereby ADSCs were transfected with miR-214-5p mimic or inhibitor. qPCR and ELISA were used to record TSG-6 expression and protein abundance, respectively. qPCR data showed that TSG-6 mRNA levels were significantly downregulated in ADSCs transfected with a miR-214-5p mimic compared to the mimic control. In agreement, we also observed that TSG-6 mRNA levels were increased compared to the control when ADSCs were transfected with a miR-214-5p inhibitor. Similar observations, though less pronounced but still significantly different, were made at the TSG-6 protein level (Figure 10).

**Figure 11:**
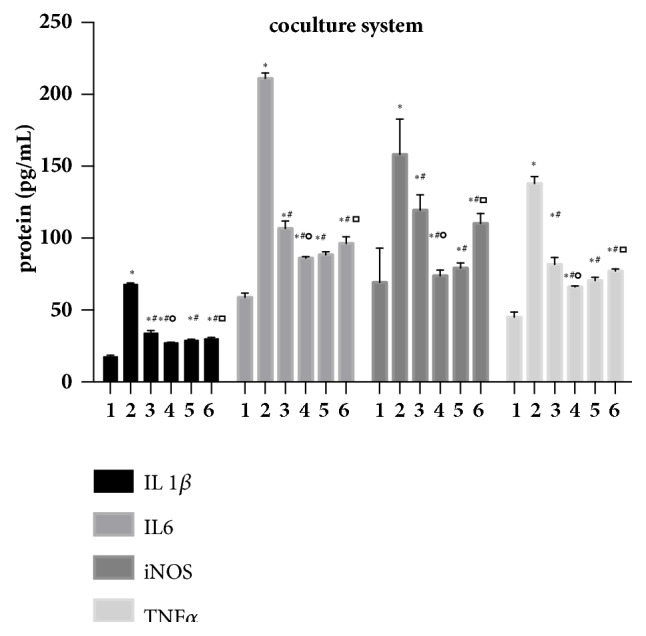
miR-214-5p negatively regulates the immunosuppressive capacity of ADSCs. Results are representative of three independent experiments using three different ADSCs cultures. There are six treatment groups in total, group 1 (BV2 microglia), group 2 (BV2 microglia+LPS), group 3 (BV2 microglia+LPS+miR214 5p-mimic ADSCs), group 4 (BV2 microglia+LPS+miR214 5p-mimic control ADSCs), group 5 (BV2 microglia+LPS+miR214 5p-inhibitor ADSCs), and group 6 (BV2 microglia+LPS+miR214 5p-inhibitor control ADSCs). IL-1*β*, IL-6, iNOS, and TNF*α* were decreased in ADSCs transfected with miR-214-5p inhibitor as compared to the inhibitor control; increased in ADSCs transfected with miR-214-5p mimic as compared to the mimic control. Comparison with group1, *∗*P<0.05; with group2, #P<0.05; with group 3, ∘P<0.05; and with group5, P<0.05.

To show that the miR-214-5p mimic and inhibitor also directly affect the immunosuppressive capacity of ADSCs, inflammatory cytokine levels in microglial cells were detected by ELISA. Data analysis showed that the expression of interleukin 1*β*, interleukin 6, iNOS, and TNF*α* was significantly elevated in LPS-induced BV2 microglia. All four cytokines in BV2 microglia were inhibited when cocultured with ADSCs for 6 h using a transwell coculture system. In addition, IL-1*β*, IL-6, iNOS, and TNF*α* were decreased in ADSCs transfected with miR-214-5p inhibitor as compared to the inhibitor control and increased in ADSCs transfected with miR-214-5p mimic as compared to the mimic control (Figure 11). These results showed that miR-214-5p inhibited the TSG-6 expression and weaken the immunosuppressive capacity of ADSCs.

## 4. Discussion

Small molecules responsible for the immunoregulatory competence of MSCs are transforming growth factor-*β* (TGF-*β*), prostaglandin E2, hepatic growth factor, epidermal growth factor, fibroblast growth factor, platelet-derived growth factor, vascular endothelial growth factor, insulin growth factor, stromal cell derived factor 1, nitric oxide, and TSG-6 [15–17].

Tumor necrosis factor-inducible gene 6 protein, also known as TNF-stimulated gene 6 protein or TSG-6, is encoded by the TNF*α*IP6 (tumor necrosis factor alpha-induced protein 6 gene). TSG-6 is a secreted protein belonging to a member of the hyaluronan-binding protein family and plays an important role in the inflammatory protease network. It interacts with a plethora of matrix associated molecules, such as aggrecan, versican, thrombospondin, pentraxin-3, and fibronectin, and subsequently affects the downstream signaling pathway and various ligand proteins. These interactions, in turn, regulate TSG-6 activity. Specifically, TSG-6 binds to the CD44 molecules and interferes with downstream nuclear factor NF-*κ*B signaling pathway to reduce the proinflammatory cytokines in the early phase of inflammation. TSG-6 is shown to improve the memory of a traumatic encephalitis model [18].

Choi et al. [19] found that MSCs treated with TNF*α* could treat a peritonitis mouse model induced by zymosan through the secretion of TSG-6. TSG-6 interferes with the CD44 molecules on the cell surface of host macrophages. Lee et al. [20] reported that TSG-6 could be a biomarker to predict the efficacy of human mesenchymal stem/progenitor cells (hMSCs) in modulating sterile inflammation in vivo. In our study, both TSG-6 mRNA and protein levels were upregulated in ADSCs after a treatment with TNF*α*, which is consistent with previous findings in bone marrow mesenchymal stem cells. Both the TNF*α* concentration and treatment duration were based on those studies referred above. The changes in TSG-6 protein abundance were smaller compared to that of mRNA and could be because protein levels were measured before reaching their maximum. Nevertheless, both observations were statistically significant between TNF*α*-treated and untreated ADSCs. To our knowledge, no study previously explored the relationship between adipose stem cells and TSG-6 proteins in vitro.

Microglia derived from the yolk sac are residential macrophages in the central nervous system during embryonic development [2]. Microglia are not uniformly distributed throughout the brain and vary between regions and species. Microglial activation results in diverse phenotypic profiles that have historically been simplified as classic or alternative forms of activation. In models of neuroinflammation, classically activated microglia have been associated with proinflammatory cytokine production and secretion of inducible nitric oxide species [21]. Alternatively activated microglia secrete anti-inflammatory cytokines and neurotrophic factors and are associated with wound healing and repair. There are two phenotypes; the M1 phenotype mainly mediates innate immune response under physiological condition to maintain homeostasis and immune surveillance. Once exposed to the inflammatory environment or mediators, the microglia are rapidly activated and release proinflammatory factors, such as TNF*α* and IL-1; the M2 phenotype [1]. Therefore, microglia play a dual role both in neuroprotection and neurotoxicity. M2 microglia secrete anti-inflammatory cytokines and neuroprotective factors, thereby protecting the brain from injury caused by oxidative stress and proinflammatory neurotransmitters. The M1 neurotoxic type on the other hand secretes proinflammatory mediators such as IL-1, TNF*α*, IL-6, and iNOS, thus inflicting damage to neurons and promoting cell death [3, 22]. In addition, the activated microglia can alter their surface molecules and initiate a more serious cascade reaction [23, 24]. Consequently, microglia need to be rigorously regulated in stroke and neurodegenerative diseases [25].

Mesenchymal stem cells (MSCs) have long been used in preclinical and clinical research [26], and stem cell therapy has become one of the most promising strategies for the treatment of various inflammatory diseases. Liu et al. found that MSCs treated with TNF*α* could inhibit the immune response in LPS-induced BV2 microglia. They also observed a reduced production of proinflammatory cytokines and that the secretory TSG-6 bound to cell surface CD44 of BV2 microglia interfered less with the downstream NF-*κ*b signaling [5]. When the gene encoding the CD44 molecules was silenced, the above was no longer observed. In addition, TSG-6 induces the expression of CD44 molecules on the cell surface. Kato et al. found that recombinant TSG-6 markedly suppressed alloreactive T cells through downregulating CD44 [27]. Many studies have shown that TSG-6 are important for MSCs in treating central nervous system diseases, such as traumatic brain injury, cerebral hemorrhage, and cerebral infarction [18, 28].

Our results show that ADSCs treated with TNF*α* express significantly more TSG-6 and have higher TSG-6 protein levels and LPS-activated BV2 cells secrete significantly less cytokines after cocultured with ADSCs treated with TNF*α*. In agreement, silencing of the TSG-6 mRNA in ADSCs using specific siRNA univocally abolished their anti-inflammatory capacity. Taken together, these data suggest that ADSCs regulate inflammation in microglia primarily by secreting TSG-6 protein.

Recently, several studies have shown that the immunoregulatory capacity of MSCs was modulated by microRNAs, including miR-146a, miR-155, and miR-181a, which directly or indirectly target the mRNAs encoding regulatory proteins in MSCs. Matysiak et al. [29] performed a differential analysis of microRNA profiles in MSCs under inflammatory and physiological conditions and found that microRNA-146a impaired the MSCs immunoregulatory capacity by directly targeting at prostaglandin E2 Synthase-2. Similarly, Xu et al. [30] found that miR-155 indirectly downregulated iNOS expression by targeting TGF*β*-activated kinase-binding protein 2 in MSCs. Liu et al. [31] found that miR-181a interfered with TGF*β* signaling pathway and altered the MSCs immunoregulatory capacity in order to maintain the immune balance.

The mechanisms behind microRNA regulation of the immunoregulatory capacity of ADSCs are yet to be elucidated. Our study obtained the microRNA profiles of TNF*α*-treated and control ADSCs through high-throughput sequencing. The expression comparison and cluster analysis between the two treatment groups indicated some differences. A total of 35 microRNAs were found to be differently expressed, 21 of which were significant, 19 were downregulated, and 16 were upregulated in ADSCs treated with TNF*α*. Specifically, miR-146a was downregulated and miR-181a was upregulated, which is consistent with previous reports [29, 31].

High-throughput sequencing (RNAseq) quantifies a dynamic range of over 8,000-fold and features good sensitivity in both low and high (saturation) ranges compared with microarray technology [11]. This technology has been applied to observe the expression of miRNAs in MSC derived from various tissues, and has identified common and tissue-specific miRNAs [32, 33]. Interpretation of the profiling and target prediction data drew attention to miR-214-5p. We believe that it might regulate TSG-6 mRNA, and the seed location could be in the 200 bp 3′UTR region.

miR-214 was discovered in HeLa cells and was shown to induce apoptosis in HeLa cells. This microRNA interferes with the Hedgehog signaling pathway and induces apoptosis in muscle cells [34]. miR-214 can regulate a variety of tumor cells and is itself regulated by a variety of signaling pathways. It has been reported that a number of transcription factor binding elements are located upstream of the miR-214 gene fragment. Lee et al. reported the role of Twist basic helix-loop-helix-transcription factor 1 (Twist-1) in regulating miR-214 transcription and found that miR-214 was increased fivefold in N2a cells transfected with adenovirus carrying Twist-1 mRNA compared to controls [35]. Further studies have shown that overexpressed miR-214 directly targeted the 3 ‘UTR region of Twist mRNA and inhibited the synthesis of Twist protein. There exists a negative feedback loop between Twist protein synthesis and miR-214 expression. Studies also showed that treatment with LPS and endoplasmic reticulum stress activated the NF-*κ*B signaling pathway and inhibited the expression of miR-199a/ miR-214 polymer in liver cancer cells. Correspondingly, inhibition of the NF-*κ*B signaling pathway increased miR-214 expression [36]. The researchers also found that miR-214 expression was upregulated in T cells stimulated by CD3 and CD28 [37].

Our study found that miR-214-5p expression dropped by 50% in ADSCs treated with TNF*α* compared with the control group. This was probably the result of the activation of the NF-*κ*B pathway in ADSCs by the proinflammatory cytokine TNF*α*, leading to an increase in NF-*κ*B, which in turn inhibited the expression of miR-214. We examined TSG-6 mRNA and protein levels in ADSCs transfected with either a miR-214-5p mimic or inhibitor. Both mRNA and protein levels were increased in ADSCs transfected with the miR-214-5p inhibitor and decreased when transfected with the mimic, indicating that miR-214-5p may directly regulate the TSG-6 mRNA. To this date, no studies have reported the role of microRNAs in regulating expression of TSG-6 mRNA and protein. The study presented herein demonstrated that TSG-6 mRNA and protein are regulated by miR-214-5p and revealed the molecular mechanisms behind the immunoregulatory capacity of ADSCs. We believe that this work lays the foundation for future clinical applications.

Our research indicates that rat ADSCs are able to regulate the immune reaction in LPS-activated microglia by TSG-6 protein. MicroRNAs are highly conserved among species. However, future work will still be needed to verify that TSG-6 expression and protein abundance in ADSCs are also regulated by microRNAs in humans. Our experiments had some limitations including the small sample size in both treatment groups. However, we believe that, because of the greater sensitivity of the RNAseq technology compared to microarray technology, we were still successful in analyzing the microRNA profiles from two treatment groups. Our study also lacked further functional analysis and pathway analysis. This will have to be addressed in future studies as the aim of this study was the identification of upstream microRNAs regulating TSG-6 expression through microRNA profiling.

## Figures and Tables

**Table 1:** The difference in microRNAs profiles between ADSCs with and without the treatment of TNFa.

Upregulation			Downregulation		
microRNA	control	experimental	microRNA	control	experimental
rno-miR-503-5p	106.43495	49.5005	rno-miR-27a-5p	54.8688	116.41815
rno-miR-214-5p	4.2359	1.3555	rno-miR-9b-3p	13.337	26.961
rno-miR-351-3p	4.95285	2.1614	rno-miR-124-3p	0	2.77845
rno-miR-335	3.4797	1.6018	rno-miR-147	0.5633	2.59875
rno-miR-1843b-5	1.042	0	rno-miR-219a-2-3p	0.4787	1.9792
rno-miR-344a-3p	0.81935	0	rno-miR-9a-3p	0.4787	1.92915
rno-miR-540-5p	0.6782	0	rno-miR-155-3p	0	1.7257
rno-miR-667-5p	0.5585	0	rno-miR-33-3p	0	1.285
rno-miR-412-5p	0.39895	0	rno-miR-203b-5p	0	0.67775
rno-miR-6319	0.39895	0	rno-miR-338-3p	0	0.50055
rno-let-7g-3p	0.39895	0			

Footer: 35 microRNAs were differentially expressed between two groups of ADSCs, 21microRNAs were significantly different.

## Data Availability

The data used to support the findings of this study are included within the article.

## Supplementary Materials

Supplementary Materials All the gene primers and amplification conditions and siRNA and miR sequences were shown in Supplementary File. Supplementary Table S1: fluorescence-conjugated antibodies used in flow cytometry analysis of ADSCs. Supplementary Table S2: 35 microRNAs were differentially expressed between two groups of ADSCs, 19 microRNAs were downregulated, and 16 were upregulated in TNFa treated ADSCs.
