# Supplementary material for: Upregulated TSG-6 Expression in ADSCs Inhibits the BV2 Microglia-Mediated Inflammatory Response
Source: Biomed Res Int. 2018 Nov 21;2018:7239181. doi: 10.1155/2018/7239181 (PMC6280241; doi:10.1155/2018/7239181)
Supplement: Supplementary Materials — All the gene primers and amplification conditions and siRNA and miR sequences were shown in Supplementary File. Supplementary Table S1: fluorescence-conjugated antibodies used in flow cytometry analysis of ADSCs. Supplementary Table S2: 35 microRNAs were differentially expressed between two groups of ADSCs, 19 microRNAs were downregulated, and 16 were upregulated in TNFa treated ADSCs. [file 7239181.f1.zip › 7239181/7239181.f1.pdf]

1. Primer sequences:

Gene	Forward (top) Reverse (bottom)	size（bp）
GAPDH	5’- CCATGTTCGTCATGGGTGTGAACCA-3’<br>5’- GCCAGTAGAGGCAGGGATGATGTTC-3’	189
TSG-6	5’- GCTACAACCCGAACGCAAAG-3’<br>5’- TACAAAGCCGTGGACATCGT-3’	251

Gene	Forward (top) Reverse (bottom)	Size（bp）
GAPDH	5’- CCATGTTCGTCATGGGTGTGAACCA-3’<br>5’- GCCAGTAGAGGCAGGGATGATGTTC-3’	189
IL-1b	5’- GCTACAACCCGAACGCAAAG-3’<br>5’- TACAAAGCCGTGGACATCGT-3’	104
IL-6	5’-CGGAGAGGAGACTTCACAGAG-3’<br>5’-CATTTCCACGATTTCCCAGA-3’	105
TNFα	5’-TATGGCTCAGGGTCCAACTC-3’<br>5’-GGAAAGCCCATTTGAGTCCT-3’	126
iNOS	5’-CCAGAAGCAGAATGTGACCA-3’<br>5’-GGACCAGCCAAATCCAGTC-3’	119

Reaction condition：
95°C 3 min；95℃ 5s，60℃ 30s，72℃ 30 s；40 cycles, 95°C 10s, 65℃ to 95.0°C

2. miR-214-5p primer sequence

Gene	Forward (top) Reverse (bottom)
U6 primer	5’-CGCTTCGGCAGCACATATAC-3’<br>5’-AAATATGGAACGCTTCACGA-3’
miR-214-5p loop primer	5’-GTCGTATCCAGTGCAGGGTCCGAGGTATTCG CACTGGATACGAC AGACACAT
miR-214-5p antisense strand primer	F primer：TGCGCAGAGTTGTCATG<br>R primer：CCAGTGCAGGGTCCGAGGTATT

Reaction condition：
50°C 2 min, 95°C 10min；95°C 30 sec ，60°C 30 sec，40 cycles

3. TSG-6-siRNA sequence:
Target sequence: CCTTGAAGTTTCTGAGTGA
sense strand (5'-3') 5’ CCUUGAAGUUUCUGAGUGA dTdT 3’
antisense strand (3'-5') 3’ dTdT GGAACUUCAAAGACUCACU 5'

4. miR-214-5p sequence: agaguugucaugugucu
miR-214-5p mimic is a double strand consisting of miR-214-5p sequence and its complementary sequence;
miR-214-5p inhibitor is a single strand consisting of the complementary sequence of miR-214-5p sequence.
