# Supplementary material for: Upregulated TSG-6 Expression in ADSCs Inhibits the BV2 Microglia-Mediated Inflammatory Response
Source: Biomed Res Int. 2018 Nov 21;2018:7239181. doi: 10.1155/2018/7239181 (PMC6280241; doi:10.1155/2018/7239181)
Supplement: Supplementary Materials — All the gene primers and amplification conditions and siRNA and miR sequences were shown in Supplementary File. Supplementary Table S1: fluorescence-conjugated antibodies used in flow cytometry analysis of ADSCs. Supplementary Table S2: 35 microRNAs were differentially expressed between two groups of ADSCs, 19 microRNAs were downregulated, and 16 were upregulated in TNFa treated ADSCs. [file 7239181.f1.zip › 7239181/supplement table S2.docx]

| miRNA_ID | miRNA normalized expression  mean value  control ADSCs | | up/down | \|log2(foldchange)\| | P-value | Significance  Lable |
| --- | --- | --- | --- | --- | --- | --- |
| rno-miR-335 | 3.4797 | 1.6018 | down | 1.119268906 | 0.002770892 | ** |
| rno-miR-338-3p | 0 | 0.50055 | up | 12.28929847 | 0 | ** |
| rno-miR-344a-3p | 0.81935 | 0 | down | 13.00026414 | 0.005146322 | ** |
| rno-miR-351-3p | 4.95285 | 2.1614 | down | 1.196292839 | 0.00084876 | ** |
| rno-miR-9a-3p | 0.4787 | 1.92915 | up | 2.010771613 | 0.003597401 | ** |
| rno-miR-27a-5p | 54.8688 | 116.41815 | up | 1.085258068 | 0 | ** |
| rno-miR-33-3p | 0 | 1.285 | up | 13.64948074 | 7.84E-05 | ** |
| rno-miR-124-3p | 0 | 2.77845 | up | 14.76199266 | 1.49E-09 | ** |
| rno-miR-214-5p | 4.2359 | 1.3555 | down | 1.643843418 | 7.55E-05 | ** |
| rno-miR-219a-2-3p | 0.4787 | 1.9792 | up | 2.047723694 | 0.003597401 | ** |
| rno-miR-412-5p | 0.39895 | 0 | down | 11.96199223 | 0 | ** |
| rno-miR-540-5p | 0.6782 | 0 | down | 12.72749507 | 0.005146322 | ** |
| rno-miR-503-5p | 106.43495 | 49.5005 | down | 1.104456963 | 1.13E-46 | ** |
| rno-miR-147 | 0.5633 | 2.59875 | up | 2.205842476 | 0.000163513 | ** |
| rno-miR-667-5p | 0.5585 | 0 | down | 12.44734157 | 0.009982122 | ** |
| rno-miR-203b-5p | 0 | 0.67775 | up | 12.72653749 | 0.006067272 | ** |
| rno-miR-9b-3p | 13.337 | 26.961 | up | 1.015439822 | 3.86E-11 | ** |
| rno-miR-6319 | 0.39895 | 0 | down | 11.96199223 | 0 | ** |
| rno-miR-155-3p | 0 | 1.7257 | up | 14.07489406 | 2.09E-06 | ** |
| rno-let-7g-3p | 0.39895 | 0 | down | 11.96199223 | 0 | ** |
| rno-miR-1843b-5p | 1.042 | 0 | down | 13.34706766 | 0.000363574 | ** |
| rno-miR-331-5p | 0.4388 | 0 | down | 12.09934781 | 0.019361935 | * |
| rno-miR-337-3p | 0 | 0.6007 | up | 12.55242895 | 0.012524143 | * |
| rno-miR-342-5p | 2.64135 | 1.06505 | down | 1.310354322 | 0.011390933 | * |
| rno-miR-146a-3p | 0 | 0.53255 | up | 12.37870127 | 0.012524143 | * |
| rno-miR-223-3p | 0.5186 | 0 | down | 12.34040649 | 0.019361935 | * |
| rno-miR-196b-3p | 0.4787 | 0 | down | 12.22490609 | 0.019361935 | * |
| rno-miR-541-3p | 0.4388 | 0 | down | 12.09934781 | 0.019361935 | * |
| rno-miR-500-5p | 0.4388 | 0 | down | 12.09934781 | 0.019361935 | * |
| rno-miR-3576 | 0.4388 | 1.6018 | up | 1.868058594 | 0.023787271 | * |
| rno-miR-6315 | 0 | 0.65075 | up | 12.66788769 | 0.012524143 | * |
| rno-miR-6318 | 0 | 0.58095 | up | 12.50419829 | 0.012524143 | * |
| rno-miR-3072 | 0.5186 | 0 | down | 12.34040649 | 0.019361935 | * |
| rno-miR-1247-3p | 0.6145 | 0 | down | 12.5851973 | 0.019361935 | * |
| rno-miR-770-3p | 0.6383 | 1.32685 | up | 1.055698731 | 0.160448681 |  |

Table S2: Differential miRNA screening criteria: In the experiment, the results showed the direction and extent of change, log2 (Fold Change) absolute value greater than or equal to 1, P <0.01 for the significant difference, log2 (Fold Change) absolute value greater than or equal to 1, P<0.05 for the general significant difference, P≥0.05 for the general difference. **means significant difference and *means general significant difference.
