# Supplementary material for: Upregulated TSG-6 Expression in ADSCs Inhibits the BV2 Microglia-Mediated Inflammatory Response
Source: Biomed Res Int. 2018 Nov 21;2018:7239181. doi: 10.1155/2018/7239181 (PMC6280241; doi:10.1155/2018/7239181)
Supplement: Supplementary Materials — All the gene primers and amplification conditions and siRNA and miR sequences were shown in Supplementary File. Supplementary Table S1: fluorescence-conjugated antibodies used in flow cytometry analysis of ADSCs. Supplementary Table S2: 35 microRNAs were differentially expressed between two groups of ADSCs, 19 microRNAs were downregulated, and 16 were upregulated in TNFa treated ADSCs. [file 7239181.f1.zip › 7239181/supplement table S1.docx]

| Table S1:  Fluorescence-conjugated antibodies used in flow cytometry analysis of ADSCs. |
| --- |
|  |

Primary antibody Company/catalog #

CD29-FITC ebioscience/ 85-11-0291-80 FITC

CD34-FITC Santa Cruz/sc-7324 FITC

CD45-FITC ebioscience / 85-11-0461-80 FITC
